# Liver Regional Oxygen Saturation in Preterm Infants with Patent Ductus Arteriosus Status

**DOI:** 10.3390/biomedicines14020361

**Published:** 2026-02-04

**Authors:** Minsoo Kim, Moon-Yeon Oh, Sol Kim, Yumi Seo, Jeongmin Shin, Sook Kyung Yum

**Affiliations:** Department of Pediatrics, College of Medicine, The Catholic University of Korea, Seoul 06591, Republic of Korea

**Keywords:** near-infrared spectroscopy, liver regional oxygen saturation, preterm infants, hemodynamically significant PDA

## Abstract

**Background/Objectives:** Near-infrared spectroscopy measures regional oxygen saturation (RSO_2_) in target tissues. Cerebral and renal RSO_2_ are associated with hemodynamically significant patent ductus arteriosus (PDA) in preterm infants. The aim of this study was to determine whether liver RSO_2_ is associated with PDA status. **Methods**: Preterm infants born before 32 weeks of gestation and/or weighing <1.5 kg were enrolled. Cerebral, renal, and liver RSO_2_ values were recorded on day 2 (D2; 48–72 h), day 7 (D7; 7 ± 2 days), and day 14 (D14; 14 ± 3 days), along with the corresponding point-of-care echocardiographic findings. **Results:** Of the forty preterm infants enrolled (mean ± SD gestational age, 28.8 ± 2.2 weeks; birthweight, 1209.9 ± 364.2 g), 22 (55.0%) had spontaneous PDA closure, 18 (45.0%) underwent pharmacological closure, and 4 (10.0%) required surgical closure. Cerebral, renal, and liver RSO_2_ values were significantly lower in infants with PDA than those without. Liver RSO_2_ was significantly lower in the presence of PDA at D7 (68.19 ± 13.24 vs. 82.14 ± 5.58, *p* < 0.001) and D14 (59.33 ± 12.06 vs. 80.0 ± 6.48, *p* < 0.001). Liver RSO_2_ showed a strong inverse association with a higher LA/Ao ratio (β −38.71, 95%CI −55.03 to −22.40, *p* <0.001) in the linear mixed model analysis. **Conclusions:** Liver RSO_2_ was associated with different PDA statuses. Future studies exploring the potential utility of liver RSO_2_ as an adjunct parameter for detecting and guiding the management of hemodynamically significant PDA in preterm infants may be warranted.

## 1. Introduction

The ductus arteriosus is a vascular connection between the pulmonary artery and the descending aorta that normally closes within several hours to days after birth. However, ductal closure is often delayed in preterm infants. The prevalence of patent ductus arteriosus (PDA) in extremely preterm infants is approximately 21% at 27–28 weeks of gestation, 64% at 25–26 weeks, and 93% at 23–24 weeks of gestation [1]. Hemodynamically significant PDA (hsPDA) can impair organ perfusion depending on the magnitude of left-to-right shunting and is associated with several morbidities in preterm infants, including pulmonary edema, pulmonary hemorrhage, pulmonary hypertension, acute kidney injury, and intraventricular hemorrhage [2]. Therefore, timely management is essential to minimize these complications.

Echocardiography is the gold standard for detecting PDA; however, it is limited by inter-operator variability and the inability to monitor dynamic changes continuously [3]. Near-infrared spectroscopy (NIRS) is a potential adjunctive tool. NIRS provides indirect information on organ perfusion by assessing changes in cerebral and renal regional oxygen saturation (RSO_2_) and fractional tissue oxygen extraction, thereby aiding in the evaluation of hsPDA [4,5]. Its advantages include continuous real-time monitoring, non-invasiveness, and operator independence [6]. Several studies have reported an association between cerebral NIRS measurements and hsPDA [7,8].

Splanchnic circulation is primarily regulated by the sympathetic nervous system, with vasoconstriction mediated by angiotensin II [9]. Compared with the brain and kidneys, splanchnic organs possess less developed autoregulatory capacity and lack effective volume-preserving mechanisms, making them more susceptible to ischemia [10]. Consequently, splanchnic tissues may exhibit early changes in RSO_2_ in the presence of hsPDA. As a solid, relatively immobile organ, the liver may reflect splanchnic oxygenation more accurately than other organs [11], a concept validated in experimental animal models [12].

Therefore, in this study, we examined the relationship between liver RSO_2_ and PDA in preterm infants, focusing on the liver as a representative splanchnic organ. We aimed to determine whether liver RSO_2_ shows meaningful changes that could facilitate earlier detection of hsPDA compared with cerebral or renal RSO_2_. Specifically, the primary objective was to observe the trajectory of liver RSO_2_ changes in patients with hsPDA, in comparison to cerebral and renal RSO_2_. The secondary objective was to evaluate the RSO_2_ levels with regard to certain echocardiographic parameters reflecting hsPDA. We hypothesized that liver RSO_2_ would be (i) lower at earlier time points than its cerebral and renal counterparts and (ii) further reduced in the presence of more severe hypoperfusion associated with hsPDA.

## 2. Materials and Methods

### 2.1. Study Design and Enrollment

This prospective cohort study was conducted in a level IV neonatal intensive care unit (NICU). Preterm infants born before 32 weeks of gestation or with a birthweight below 1.5 kg between January 2023 and January 2025 were eligible for inclusion. The Institutional Review Board (IRB) of our institute approved the study (approval number: KC22ONSI0820 date: 16 November 2022). As this study was exploratory in nature, and reliable estimates of the expected effect size and variance for liver RSO_2_ in this context were not available, we aimed to enroll as many eligible infants as possible from the participating subunit over the study period through consecutive sampling. The infants were enrolled after obtaining written informed consent from their parents. The exclusion criteria were major congenital malformations, chromosomal or genetic abnormalities, and parental refusal to provide informed consent. Among the 67 infants who met the initial target population criteria, one infant with major congenital anomalies and six who died before enrollment were excluded. Additional exclusions were owing to parental refusal (*n* = 15) or withdrawal of informed consent (*n* = 5), resulting in a final sample of 40 infants. The patient enrollment scheme is presented in Supplementary Figure S1.

### 2.2. RSO_2_, Echocardiographic Parameters, and Clinical Data Collection Regarding PDA

Cerebral, renal, and liver RSO_2_ values were measured using near-infrared spectroscopy (INVOS^TM^ PM7100 System, Medtronic, Dublin, Ireland) at three predefined time points: day 2 (D2, 48–72 h of life), day 7 (D7, 7 ± 2 days), and day 14 (D14, 14 ± 3 days). Probe placement was standardized using anatomical landmarks and confirmed by a member of the research team present at the time of placement according to a written protocol. For cerebral measurements, the probe was placed on the frontal region above the eyebrows and below the hairline, avoiding the sagittal suture and anterior fontanelle. For renal and liver measurements, the probe was placed over the posterior flank at the level of the kidney and over the right upper quadrant below the costal margin, respectively. Light-impermeable adhesive dressings were used to secure the probe and minimize ambient light interference. The monitor was zeroed and allowed to stabilize for several minutes after probe application, and readings with obvious motion artifacts (sudden step changes or loss of signal during handling or repositioning) were excluded or re-measured after repositioning the probe. The device performed internal calibration according to the manufacturer’s specifications. At each time point, point-of-care echocardiography was performed concurrently by neonatologists trained in targeted neonatal echocardiography (M.K., M.O., and S.K.Y., with 5, 3, and 10 years of experience, respectively) using a predefined standardized protocol for PDA assessment. An Affiniti 50 echocardiography system (Philips Ultrasound, Bothell, Seattle, WA, USA) equipped with an S12-4 neonatal cardiac transducer (4–12 MHz) was used for echocardiography.

A standardized protocol for PDA assessment conducted at the same three time points as the NIRS assessments, which included exemplary images with markers specifying measurement techniques for each parameter, was developed (by S.K.Y.) and distributed to all researchers prior to enrollment. This protocol included the evaluation of ductal patency, measurement of ductal diameter, determination of shunt direction (right-to-left, bidirectional, or left-to-right), peak systolic velocity, and classification of the Doppler flow pattern. The luminal diameter of the ductus was measured in the high parasternal view at its narrowest point near the pulmonary end using the 2D image [13]. hsPDA was assessed by evaluating echocardiographic parameters suggestive of pulmonary overcirculation and systemic hypoperfusion, along with ductal size and flow velocity. The evaluated parameters included the left pulmonary artery end-diastolic velocity (LPA EDV, cm/s), left atrium-to-aortic root ratio (LA/Ao), left ventricular end-diastolic diameter (LVEDD, mm) [14], and cerebral and renal perfusion indices. Specifically, echocardiographic indicators of hsPDA included a ductal diameter ≥ 1.5 mm, as well as markers of left ventricular volume loading, such as an LPA EDV ≥ 0.2 m/s and LA/Ao ratio ≥ 1.4 [15,16].

In addition to echocardiographic indices, hsPDA was clinically characterized by the presence of PDA-related signs, including: (1) a systolic or continuous cardiac murmur; (2) bounding peripheral pulses or hyperdynamic precordial activity; (3) difficulty maintaining adequate blood pressure (e.g., hypotension unresponsive to fluid resuscitation, requiring hydrocortisone and/or vasopressor(s), defined as arterial blood pressure below the lower limit for corrected gestational age); (4) worsening respiratory status; and (5) supportive radiographic findings, such as pulmonary congestion or cardiomegaly.

The echocardiography images were reviewed by a researcher (S.K.Y.) to ensure protocol coherence and measurement consistency. Clinical parameters, including blood pressure, respiratory symptoms, level of respiratory support, blood urea nitrogen, serum creatinine, and hourly urine output, were assessed on the same day as the NIRS and echocardiographic evaluations. For laboratory parameters, the nearest value within 3 days of the NIRS measurements was used.

The primary treatment strategy for PDA in the enrolled preterm infants included the initiation of pharmacological treatment when echocardiography demonstrated moderate-to-severe hsPDA associated with systemic hypoperfusion or cerebral hemodynamic instability [17,18]. Ibuprofen was used as the first-line agent [19], whereas paracetamol was administered to infants with contraindications to ibuprofen, such as active bleeding, severe intraventricular hemorrhage, necrotizing enterocolitis (stage 2 or 3), or oliguria [20]. If paracetamol proved ineffective and ibuprofen was no longer contraindicated, ibuprofen was administered as a second-line treatment. Surgical ductal closure was considered in infants with persistent symptoms and no response to medical therapy [21].

### 2.3. Other Clinical Data Collection

Baseline neonatal and maternal characteristics were recorded, including gestational age (weeks), birthweight (g), sex, mode of delivery (cesarean section or vaginal), 1- and 5 min Apgar scores, maternal age (years), maternal diabetes or hypertension, histologic chorioamnionitis, plurality (singleton or multiple gestation), mode of conception (natural or assisted reproductive technology), antenatal corticosteroid administration, and the mode of respiratory support (use and duration of invasive ventilation). Data collection was performed using REDCap (https://project-redcap.org/).

### 2.4. Statistical Analysis

Descriptive statistical analyses were performed using SPSS software (version 20.0; IBM Corp., Armonk, NY, USA). Infants were classified into two groups based on the presence or absence of PDA, and their blood pressure and cerebral, renal, and liver RSO_2_ values were compared. RSO_2_ values were compared in relation to echocardiographic parameters. Continuous variables were analyzed using Student’s *t*-test for those with a parametric distribution and the Mann–Whitney *U* test for those with a non-parametric distribution. Categorical variables were assessed using the chi-squared or Fisher’s exact tests, as appropriate. To assess longitudinal associations between ductal loading and RSO_2_ values, linear mixed-effects models (LMM) were fitted with random intercepts for subjects to account for intra-patient correlation and unbalanced repeated measurements using R version 4.5.2 (R Foundation for Statistical Computing, Vienna, Austria). Time (D2, D7, and D14) was incorporated as a categorical fixed effect, and each echocardiographic PDA index was entered into a separate model as the primary predictor. All models included gestational age, invasive ventilation, and mean blood pressure (BP) at the assessment time point as covariates. The covariates were chosen to represent factors that would potentially affect regional oxygenation, in the context of organ functional maturity in premature infants, respiratory condition necessitating a relatively higher magnitude of support, and the clinical effect of hsPDA as well as the result of vasopressor use, respectively. Additional respiratory and hemodynamic variables (e.g., fraction of inspired oxygen, vasopressor use, etc.) were not included in the final models to reduce overfitting and collinearity in this small cohort. Missing data regarding RSO_2_, PDA variables, and covariates were not imputed (missingness of PDA-related echocardiographic indices is presented in Supplementary Table S1). Instead, all linear mixed-effects models were fitted using restricted maximum likelihood, which uses all available observations under a missing-at-random assumption (MAR) for the longitudinal outcomes. RSO_2_ values were analyzed on the original percentage scale, without transformation, and model estimates are reported as absolute percentage-point differences in RSO_2_. Effect estimates are presented as regression coefficients with 95% confidence intervals (CIs), and *p*-values < 0.05 were considered statistically significant for all analyses.

## 3. Results

Forty preterm infants were enrolled in the study, and 116 echocardiographic assessments were performed. The mean gestational age at birth was 28.8 ± 2.2 weeks, and the mean birthweight was 1209.9 ± 364.2 g. The other baseline characteristics of the enrolled infants are presented in Table 1.

Among the 40 infants, 22 (55.0%) achieved spontaneous closure of the PDA, 18 (45.0%) underwent pharmacological closure, and 4 (10.0%) underwent surgical closure. All surgical closures were performed as secondary interventions after unsuccessful medical closure. The mean postnatal day and postmenstrual age at treatment initiation were 4.8 ± 2.6 days and 28.3 ± 2.6 weeks, respectively (Table 2). On postnatal days 2, 7, and 14 (D2, D7, and D14, respectively), ductal patency was observed in 33 (82.5%), 17 (43.6%), and 10 (27.0%) infants. The mean ductal diameter was greater at D14 than at D2 or D7 (1.78 vs. 1.66 and 1.62 mm, respectively). Similar trends were observed for LPA EDV and LA/Ao.

When cerebral, renal, and liver RSO_2_ values were compared between the PDA and no-PDA groups at different time points, all measurements were significantly lower in the presence of PDA (Table 3). When PDA was present at any time point during hospitalization, cerebral RSO_2_ was significantly lower (74.22 ± 10.14% vs. 80.47 ± 7.91%, *p* < 0.001), as were renal RSO_2_ (65.68 ± 14.29% vs. 72.51 ± 12.88%, *p* = 0.005) and liver RSO_2_ (65.32 ± 15.91% vs. 71.30 ± 10.62%, *p* = 0.010). Liver RSO_2_ was significantly lower in infants with PDA at D7 (68.19 ± 13.24 vs. 82.14 ± 5.58, *p* < 0.001) and D14 (59.33 ± 12.06 vs. 80.0 ± 6.48, *p* < 0.001). Systolic, diastolic, and mean BP values were significantly lower in the presence of PDA than in its absence, except for systolic BP measured on D2, which showed no significant differences (Supplementary Table S2). BP values demonstrated statistically significant positive Pearson correlation coefficients with each RSO_2_ measurement (Supplementary Table S3).

**Figure 1:**
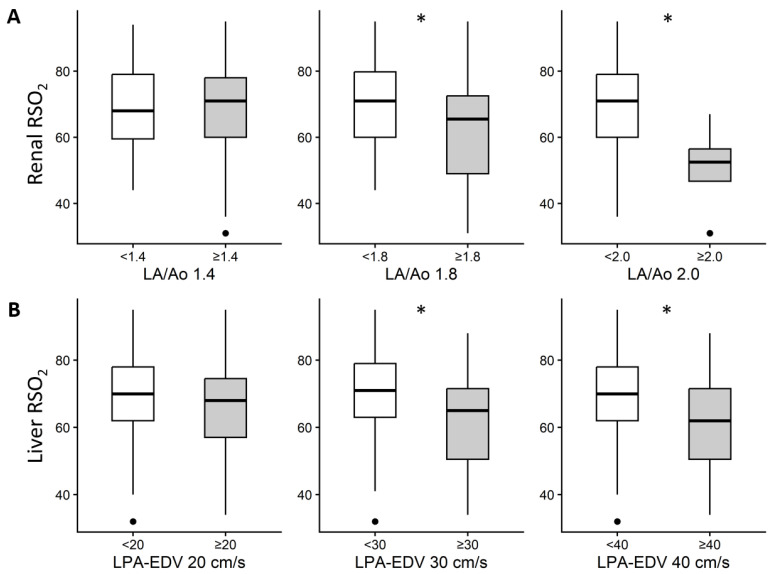
Key findings of RSO_2_ measurements with respect to echocardiographic parameters. (**A**): Renal RSO_2_ according to LA/Ao cutoffs of 1.4, 1.8, and 2.0; (**B**): Liver RSO_2_ according to LPA EDV cutoffs of 20, 30, and 40 cm/s. Boxes indicate median and interquartile range; whiskers indicate the range excluding outliers. Asterisks indicate *p* < 0.05. LPA EDV, left pulmonary artery end-diastolic velocity; LA/Ao, left atrium-to-aortic root ratio; RSO_2_, regional oxygen saturation.

The key results of the RSO_2_ analysis based on echocardiographic parameters used to assess the hemodynamic significance of PDA are illustrated in Figure 1. Detailed findings of the exploratory analyses involving specific cutoffs of all echocardiographic parameters are presented in the Supplementary Table S4. Higher LA/Ao and LPA EDV cutoffs were associated with lower renal and liver RSO_2_, respectively, with the most pronounced differences at an LA/Ao ≥ 1.8 and LPA EDV ≥ 40 cm/s.

The LMM results are presented in Table 4. Cerebral RSO_2_ showed no clear association with any PDA indices, based on the small estimate effect with 95%CI spanning negative and positive values. In contrast, for a larger PDA size, renal RSO_2_ exhibited stronger inverse associations with PDA indices. For instance, a larger PDA size was associated with a lower renal RSO_2_ (β: −18.44; 95%CI: −30.87 to −6.01), and a higher LA/Ao showed an even more prominent inverse association (β: −41.37; 95%CI: −61.06 to −20.67). For liver RSO_2_, a higher LA/Ao ratio showed a strong inverse association (β: −38.71; 95%CI: −55.03 to −22.40). Among the covariates, D14 (versus D2), invasive ventilation, and lower mean BP were associated with lower renal and liver RSO_2_ in some models. To account for the potential influence of PDA treatment timing, a binary indicator of prior treatment status was included in the model; this covariate did not materially affect the LA/Ao-renal (estimate = −39.90; 95% CI: −55.50 to −19.80) or liver RSO_2_ association (estimate = −37.82; 95% CI: −50.84 to −20.96). As a sensitivity analysis, we repeated the LMMs in a complete-case dataset restricted to observations without missing RSO_2_, echocardiographic, or covariate data, which yielded effect estimates similar in direction and magnitude to the primary values.

## 4. Discussion

We investigated the association between hsPDA and NIRS-derived RSO_2_ in the liver. Although previous studies have explored the relationship between hsPDA and NIRS parameters, to the best of our knowledge, this study is the first to specifically report liver oxygenation in the context of hsPDA, representing a unique contribution to the field.

Despite advances in neonatal care, determining the optimal timing of PDA interventions remains challenging. Echocardiography is the gold standard for hsPDA diagnosis; however, it is limited by operator dependency and technical difficulties in extremely preterm infants [3]. In contrast, NIRS is noninvasive, simple, and allows continuous bedside monitoring [4]. Although cerebral NIRS provides direct information on cerebral perfusion, its application in preterm infants undergoing noninvasive ventilation is often limited by the difficulty of sensor placement beneath the headgear. Similarly, applying renal NIRS in extremely preterm infants may be challenging because sensor placement on the dorsal surface requires repositioning, which can be burdensome for caregivers owing to fragile skin and the risk of cerebral hemodynamic fluctuation. Although intestinal NIRS can theoretically provide a more direct measure of splanchnic perfusion, its accuracy is limited by several factors, including the presence of multiple intra-abdominal organs (such as the bladder and ureters), bowel peristalsis, and interference from high biliverdin content in the meconium or transitional stools, which affects light absorption [22,23]. In this context, we speculated that liver NIRS may serve as a practical alternative for representing splanchnic perfusion and as a surrogate marker of impaired organ perfusion, in addition to conventional PDA assessment [24].

In our study, liver RSO_2_, as well as cerebral and renal RSO_2_ values, were significantly lower in infants with PDA at certain assessment time points. This finding supports the potential applicability of liver NIRS for detecting PDA-related organ hypoperfusion alongside cerebral or renal RSO_2_ values, which have been more frequently reported in previous studies, including those involving preterm infants [7,8]. However, these RSO_2_ values represent only descriptive snapshots at predetermined time points, and potential confounders, such as postnatal maturation and concurrent clinical management, including fluid therapy, specific ventilator support mode or settings, vasopressor use, transfusion, etc., were not fully accounted for. Therefore, the observed differences in RSO_2_ values per se should not be interpreted as resulting from a direct cause-and-effect relationship with hsPDA. In addition, the clinical significance of modest (5–10%) differences in RSO_2_ remains uncertain. Previous studies have reported that reductions in renal RSO_2_ below 66% or cerebral RSO_2_ below 55–60% in preterm infants are associated with hsPDA and potential organ hypoperfusion [25,26]. However, smaller changes of less than 5% are unlikely to reflect clinically meaningful perfusion impairment and should be interpreted cautiously within the broader clinical context, including factors such as blood pressure, anemia, carbon dioxide levels, and glucose levels. This is particularly true for liver RSO_2_ where validated thresholds do not exist in the previous literature nor have they been generated in this study. In other words, the clinical advantage of liver NIRS lies in its role as an easily applicable and continuous bedside monitoring tool that may provide complementary information on hemodynamic impairment associated with hsPDA, that would prompt further evaluation for diagnosis and treatment, rather than as a stand-alone diagnostic criterion.

Some unexpected findings were observed in this study. We initially hypothesized that liver NIRS would be more sensitive to systemic hypoperfusion because perfusion of solid organs is more strongly influenced by the sympathetic nervous system, with less autoregulatory capacity and volume preservation than that of the brain or kidneys [10]. However, the decrease in liver RSO_2_ did not necessarily occur earlier than that in other affected organs, such as the brain and kidneys. One possible explanation for this is the dual blood supply to the liver. Hepatic perfusion is maintained by both the portal vein and hepatic artery; therefore, a reduction in mesenteric inflow due to PDA may be compensated for by an increase in hepatic arterial flow, thereby preserving RSO_2_ for a certain period [11]. This compensatory mechanism may partially explain the differences in liver RSO_2_ according to the degree of left heart loading in the current study.

We also expected that the liver RSO_2_ value would continue to decrease in the setting of a more prominent left heart load. Although RSO_2_ values across multiple organs, including the liver, showed significant differences when stratified by smaller PDA sizes (<1.5 mm and <1.75 mm), no such differences were observed when the groups were divided using a PDA size cutoff of 2 mm. These findings suggest that the hemodynamic burden imposed by PDA may not be solely determined by its diameter. Rather, the degree of shunt loading may vary regardless of ductal size [27]. Therefore, RSO_2_ levels in different organs may not consistently decrease with increasing PDA size alone (Table 4). Notably, these findings imply that NIRS is sensitive enough to detect changes in organ perfusion that are not readily discernible by ductal size alone on echocardiography, underscoring its potential utility in assessing PDA severity in clinical practice.

Liver RSO_2_ values were significantly reduced in infants with higher LPA EDV thresholds (>30 and >40 cm/s), but not in those with lower thresholds (>20 cm/s). Additionally, liver RSO_2_ decreased as the LA/Ao ratio increased. Based on the LMM results adjusted for time-varying effects and covariates, liver RSO_2_ showed a strong inverse association with the LA/Ao ratio. This finding may explain why liver RSO_2_ values further decreased with progressive PDA severity, particularly when assessed by LA/Ao ratio, rather than by ductal size. This finding aligns with those of the study by Schat et al., which reported that liver NIRS did not discriminate between infants with and without necrotizing enterocolitis (NEC) but could differentiate uncomplicated from complicated NEC [28]. These results suggest that changes in liver RSO_2_ may become more apparent or prominent in the setting of severe hypoperfusion. In addition, invasive ventilation and mean BP showed significant negative and positive associations, respectively, with liver RSO_2_, implying greater hemodynamic vulnerability of the liver compared with other organs. Indeed, the degree of respiratory and hemodynamic support forms part of the clinical severity criteria for hsPDA, as described by McNamara et al. [18]. Therefore, monitoring liver RSO_2_ in preterm infants, particularly those requiring higher levels of respiratory support, especially invasive ventilation or vasopressors, may be useful. These findings may prompt earlier or repeated echocardiographic assessment.

Notably, RSO_2_ in all three regions showed a negative association with time, which is in line with the findings of McNeill et al. [29], who reported a decreasing tendency for cerebral and renal RSO_2_ over the first three postnatal weeks in preterm infants. To what extent this pattern reflects maturational increases in oxygen consumption in our study is unclear, because factors such as changes in fluid status, hemoglobin concentration, adjustments in respiratory support, carbon dioxide levels, vasopressor use, exposure to nephrotoxic medications [30], and feeding status [31] were not systematically captured in our models owing to the limited sample size. However, the larger time-related decreases in liver RSO_2_ (β estimate) suggest that this vascular bed may be more susceptible to evolving hemodynamic and metabolic stresses in this population, consistent with our a priori hypothesis.

The limitations of our study include its relatively small sample size and single-institution setting. As this was a prospective observational study without randomization or intervention, we adopted a consecutive sampling method to include as many eligible participants as possible during the study period. Additionally, because multiple comparisons were performed across organs, time points, and echocardiographic indices, the risk of false-positive findings cannot be completely excluded. However, our conclusions are based on the magnitude and consistency of the estimated associations (e.g., the inverse relationship between liver RSO_2_ and LA/Ao) rather than on isolated *p*-values. Large-scale studies with appropriately calculated statistical power are necessary to validate and generalize our findings and to inform future research involving interventional analyses and outcome assessments. In addition, the timing of the assessments was defined within relatively broad windows (e.g., D2: 48–72 h of life). This approach reflected the practical need to account for the availability of researchers trained in targeted neonatal echocardiography, which inevitably requires flexibility in scheduling. Because echocardiographic assessments were performed by three neonatologists, a potential for variability in the measurements was present. Nonetheless, echocardiography was performed according to a protocol with predefined standards and sample images for each parameter (produced by S.K.Y.) that were distributed to the neonatologists before study initiation, and all three neonatologists had at least 3 years of experience in targeted echocardiography. Furthermore, the obtained images were reviewed by the most experienced investigator to ensure internal consistency of measurements. However, because echocardiographic assessments were performed by three neonatologists, some degree of inter-observer variability cannot be excluded, and formal intra-or inter-observer reliability testing (e.g., intraclass correlation coefficients) was not performed in this study, which represents an additional limitation that should be addressed in future work. Furthermore, although some clinically relevant covariates were incorporated into the LMM analyses, more detailed potential confounders, including the hemodynamic and metabolic factors mentioned above, were not included because of the small sample size. Additionally, our inference relies on the plausibility of the MAR assumption for missing data, and more extensive sensitivity analyses will be required in larger cohorts. Future studies should incorporate more variables to appropriately adjust for confounding factors including concurrent treatments (e.g., fluid therapy, transfusions, vasopressor use, etc.) in order to more robustly elucidate the potential impact of these unmeasured factors in the present study. Finally, because the infants included in our study did not develop NEC, the association between liver RSO_2_ and NEC, which could occur in the setting of mesenteric hypoperfusion, was not analyzed. Altogether, future studies aiming to verify and extend our findings will require a prospective design and a larger, multicenter cohort. Such studies should minimize inter-operator variability in echocardiographic assessments and incorporate more comprehensive clinical data.

## 5. Conclusions

In this exploratory study, we provide initial reference data on NIRS-derived regional, particularly liver, RSO_2_ in preterm infants with PDA. Liver RSO_2_ was significantly lower in the presence of PDA, as identified by specific echocardiographic parameters reflecting left heart loading. However, this finding does not establish a direct cause-and-effect relationship. Further studies with predefined objectives are needed to assess the clinical utility of this modality and to identify cutoffs that can differentiate between PDA that will not require treatment, PDA likely to require medical therapy, and PDA that is likely to require surgical ligation. Future studies incorporating additional clinically relevant confounders are warranted to further validate the usefulness of liver RSO_2_ as an auxiliary parameter and to suggest potentially actionable thresholds for detecting and assessing the severity of hsPDA, and guiding therapy in preterm infants.

## Figures and Tables

**Table 1:** Demographics of the enrolled infants.

	Number (%) or Mean ± SD
**Gestational age (weeks)**	28.8 ± 2.2
**Birthweight (grams)**	1209.9 ± 364.2
**Male sex**	17 (42.5)
**Singleton**	28 (70.0)
**Cesarean delivery**	39 (97.5)
**1 min Apgar**	4.4 ± 1.6
**5 min Apgar**	7.0 ± 1.9
**Mother age (years)**	35.6 ± 4.0
**Assisted reproduction therapy**	10 (25.0)
**PPROM**	12 (30.0)
**Oligohydramnios**	3 (7.5)
**Histologic chorioamnionitis**	8 (20.0)
**Maternal diabetes**	6 (15.0)
**Maternal hypertension**	10 (25.0)
**Antenatal corticosteroid administration**	
Any	39 (97.5)
Complete	23 (57.5)
**Respiratory support**	
Invasive ventilation	19 (47.5%)
Duration of invasive ventilation (days)	8.8 ± 7.4

**Table 2:** Trajectory of ductal patency and echocardiographic findings.

				N (%) or Mean ± SD	
**PDA management**					
		Spontaneous closure		22 (55.0)	
		Medical treatment		18 (45.0)	
		Ibuprofen only		6 (33.3) *	
		Paracetamol only		9 (50.0) *	
		Both		3 (16.7) *	
		Surgical treatment		4 (10.0)	
		Primary		0 (0.0) *	
		Secondary		4 (100.0) *	
**Chronological age at treatment initiation (days)**				4.8 ± 2.6	
**PMA at treatment initiation (weeks)**				28.3 ± 2.6	
**Echocardiographic assessment**		D2	D7		D14
**Hour of life at assessment** **^a^**		67.7 [54.1–71.3]	8 [7–9]		14 [14–15]
**PMA (weeks)**		29.3 ± 2.2	29.9 ± 2.3		30.9 ± 2.2
**Prior PDA treatment**					
	Medical	0	15		3 ^b^
	Surgical	0	0		1
**Ductal patency**		33 (82.5)	17 (43.6) ^c^		10 (27.0) ^d^
**PDA size (mm) ^c^**		1.66 ± 0.53	1.62 ± 0.58		1.78 ± 0.39
**LPA end-diastolic velocity (cm/s) ^a,c^**		21.20 [13.85–35.95]	24.25 [6.80–46.58]		30.25 [23.7–35.95]
**LA/Ao ^a^**		1.59 ± 0.27	1.60 ± 0.39		1.83 [1.49–1.89]
**LVEDD (mm) ^a^**		13.66 ± 2.37	13.48 ± 2.75		13.40 [12.68–14.70]

* N (%) in the medical treatment patient group or surgical treatment group, as appropriate. ^a^ Analyzed using the Mann–Whitney *U* test and presented as median [interquartile range]. ^b^ PDA treatment between D7 and D14 assessments. ^c^ Analyzed only in patients with ductal patency. ^d^ Analysis of 39 patients with D7, and 37 patients with D14. PDA, patent ductus arteriosus; PMA, postmenstrual age; LPA, left pulmonary artery; LA/Ao, left atrium-to-aortic root ratio; LVEDD, left ventricular end-diastolic diameter.

**Table 3:** RSO_2_ measurements were compared according to ductal patency assessed by echocardiography at different time points.

	PDA	No PDA	*p*-Value
**Ductal patency at any time point ^a^**	60	56	-
Cerebral RSO_2_	74.22 ± 10.14	80.47 ± 7.91	<0.001
Renal RSO_2_	65.68 ± 14.29	72.51 ± 12.88	0.005
Liver RSO_2_	65.32 ± 15.91	71.30 ± 10.62	0.010
**Ductal patency at D2 Echo ^b^**	33	7	-
Cerebral RSO_2_	78.56 ± 7.82	87.71 ± 7.34	0.004
Renal RSO_2_	69.98 ± 11.55	77.86 ± 15.95	0.065
Liver RSO_2_	70.41 ± 14.69	73.0 ± 6.95	0.327
**Ductal patency at D7 Echo ^b^**	17	22	-
Cerebral RSO_2_	76.23 ± 9.57	85.71 ± 5.71	0.008
Renal RSO_2_	68.68 ± 14.76	74.14 ± 11.61	0.183
Liver RSO_2_	68.19 ± 13.24	82.14 ± 5.58	<0.001
**Ductal patency at D14 Echo ^b^**	10	27	-
Cerebral RSO_2_	72.17 ± 9.88	77.60 ± 6.80	0.124
Renal RSO_2_	63.67 ± 15.14	75.80 ± 8.20	0.046
Liver RSO_2_	59.33 ± 12.06	80.0 ± 6.48	<0.001

^a^ All assessed cases, at D2, D7, and D14, are counted. ^b^ Number of infants. D2, day 2, D7, day 7, D14, day 14; PDA, patent ductus arteriosus; RSO_2_, regional oxygen saturation.

**Table 4:** LMM estimates for the association between echocardiographic PDA indices and cerebral, renal, and liver RSO_2_ ^a^.

RSO_2_	PDA Index and Covariates	Estimate	95%CI	*p*-Value
**Cerebral**	PDA size	4.24	−3.87 to 12.34	0.287
	LA/Ao	−6.77	−22.37 to 8.83	0.365
	LPA-EDV	−0.03	−0.25 to 0.18	0.763
**Renal**	PDA size	−18.44	−30.87 to −6.01	0.006
	LA/Ao	−41.37	−62.36 to −20.37	0.002
	LPA-EDV	−0.46	−0.77 to −0.15	0.005
**Liver**	PDA size	−9.03	−22.09 to 4.03	0.164
	LA/Ao	−38.71	−55.03 to −22.40	<0.001
	LPA-EDV	−0.21	−0.54 to 0.12	0.198

^a^ The models were adjusted for gestational age, invasive ventilation, and mean BP. | BP, blood pressure; PDA, patent ductus arteriosus; CI, confidence interval; LPA EDV, left pulmonary artery end-diastolic velocity; LA/Ao, left atrium-to-aortic root ratio; LMM, linear mixed-effects models; RSO_2_, regional oxygen saturation.

## Data Availability

The data that support the findings of this study are available from the corresponding author, only upon reasonable request.

## Supplementary Materials

The following supporting information can be downloaded at: https://www.mdpi.com/article/10.3390/biomedicines14020361/s1, Figure S1: Patient enrolment scheme; Table S1: Number of missing indices by time; Table S2: Blood pressure values compared according to the PDA at different assessment time points; Table S3: Pearson correlation coefficients between blood pressure values and organ-specific RSO_2_ levels; Table S4: RSO_2_ measurements with respect to echocardiographic parameters.

## Abbreviations

The following abbreviations are used in this manuscript:

RSO_2_	Regional oxygen saturation
PDA	Patent ductus arteriosus
hsPDA	Hemodynamically significant patent ductus arteriosus
NIRS	Near-infrared spectroscopy
NICU	Neonatal intensive care unit
D2	Day 2
D7	Day 7
D14	Day 14
LA/Ao	Left atrium-to-aorta
LPA EDV	Left pulmonary artery end-diastolic velocity
LVEDD	Left ventricular end-diastolic diameter
LMM	Linear mixed-effects models
BP	Blood pressure
CI	Confidence intervals
