# Supplementary material for: Liver Regional Oxygen Saturation in Preterm Infants with Patent Ductus Arteriosus Status
Source: Biomedicines. 2026 Feb 4;14(2):361. doi: 10.3390/biomedicines14020361 (PMC12938450; doi:10.3390/biomedicines14020361)
Supplement: Supplementary file 1 [file biomedicines-14-00361-s001.zip › biomedicines-4063526-supplementary.pdf]

Supplementary files.

Supplementary Figure S1. Patient enrollment scheme.

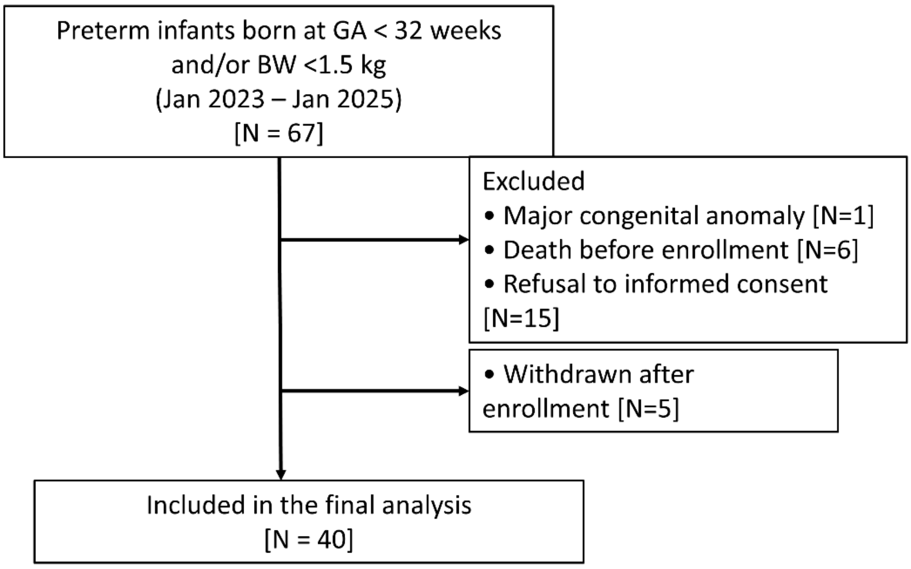

During the study period, 67 eligible infants were screened. Infants were excluded because of major congenital anomaly (n = 1), death before enrollment (n = 6), refusal of informed consent (n=15), and withdrawal of informed consent owing to clinical deterioration (n = 5). Hence, 40 infants were included in the final analysis.

BW, birthweight; GA, gestational age.

Supplementary Table S1.

Number of missing indices by time.

**Time**	**PDA status not recorded**	**PDA status recorded**			**Cerebral RSO_2**	**Renal RSO_2**	**Liver RSO_2**
		**PDA size**	**LA/Ao**	**LPA EDV**			
**D2**	0	0	1	0	1	1	1
**D7**	1	0	5	3	2	2	2
**D14**	3	0	3	3	5	5	5

PDA, patent ductus arteriosus; LPA EDV, left pulmonary artery end-diastolic velocity; LA/Ao, left atrium-to-aortic root ratio; RSO_2, regional oxygen saturation.

Supplementary Table S2.

Blood pressure values compared according to the PDA at different assessment time points.

	**PDA**	**No PDA**	**p-value**
**Ductal patency at any time point**	60	56	-
SBP (mmHg)	59.00 ± 11.00	69.50 ± 8.27	< 0.001
DBP (mmHg)	32.35 ± 8.22	41.07 ± 9.49	< 0.001
MBP (mmHg)	41.10 ± 8.57	50.30 ± 8.02	< 0.001
**Ductal patency at D-2 Echo**	33	7	-
SBP (mmHg)	59.70 ± 10.62	66.29 ± 8.73	0.067
DBP (mmHg)	32.58 ± 7.19	39.86 ± 13.55	0.023
MBP (mmHg)	41.64 ± 8.02	48.00 ± 10.33	0.039
**Ductal patency at D-7 Echo**	17	22	-
SBP (mmHg)[a]	60.00 [48.00–70.00]	70.50 [65.50–75.50]	0.002
DBP (mmHg)	32.24 ± 9.74	41.36 ± 9.34	0.003
MBP (mmHg)	40.53 ± 9.43	50.68 ± 7.55	< 0.001
**Ductal patency at D-14 Echo**	10	27	-
SBP (mmHg)	58.50 ± 11.42	69.93 ± 8.59	0.001
DBP (mmHg)	31.80 ± 9.51	41.15 ± 8.80	0.004
MBP (mmHg)	40.30 ± 9.59	50.59 ± 7.99	0.001

a. Analyzed using the Mann–Whitney *U* test and presented as median [interquartile range].

PDA, patent ductus arteriosus; SBP, systolic blood pressure; DBP, diastolic blood pressure; MBP, mean blood pressure.

Supplementary Table S3.

Pearson correlation coefficients between blood pressure values and organ-specific RSO_2 levels.

Blood pressure	Cerebral RSO_2	Renal RSO_2	Liver RSO_2
**SBP**	0.31 ($p = 0.001$)	0.35 ($p < 0.001$)	0.28 ($p = 0.003$)
**DBP**	0.23 ($p = 0.015$)	0.23 ($p = 0.015$)	0.26 ($p = 0.006$)
**MBP**	0.28 ($p = 0.003$)	0.32 ($p < 0.001$)	0.29 ($p = 0.002$)

RSO_2, regional oxygen saturation; SBP, systolic blood pressure; DBP, diastolic blood pressure; MBP, mean blood pressure.

Supplementary Table S4.

RSO_2 measurements with respect to echocardiographic parameters.

Parameter	Cutoff	Cerebral RSO_2	p-value	Renal RSO_2	p-value	Liver RSO_2	p-value
**PDA size**	≥ 1.5 mm (n=36)	73.83 ± 9.84	0.012	61.57 ± 12.95	< 0.001	62.31 ± 16.76	0.009
	< 1.5 mm(n=80)	78.70 ± 9.20		72.25 ± 13.24		70.81 ± 11.62	
	≥ 1.75 mm (n=28)	72.81 ± 10.19	0.003	59.30 ± 13.28	< 0.001	59.37 ± 15.41	< 0.001
	< 1.75 mm (n=88)	78.56 ± 9.08		71.96 ± 12.86		70.94 ± 12.26	
	≥ 2 mm (n=13)	74.08 ± 9.34	NS	61.92 ± 12.62	NS	62.33 ± 17.28	NS
	< 2 mm (n=103)	77.55 ± 9.65		69.75 ± 13.98		68.85 ± 13.41	
**LPA EDV**	≥ 20 cm/s (n=36)	73.91 ± 10.90	0.015	64.17 ± 13.25	0.016	65.49 ± 15.21	NS
	< 20 cm/s (n=80)	78.70 ± 8.73		71.08 ± 13.98		69.64 ± 13.09	
	≥ 30 cm/s (n=24)	73.65 ± 9.95	0.024	61.17 ± 13.12	0.001	61.13 ± 14.61	0.002
	< 30 cm/s (n=92)	78.11 ± 9.45		70.92 ± 13.67		70.22 ± 13.10	
	≥ 40 cm/s (n=16)	75.13 ± 11.44	NS	60.80 ± 15.21	0.016	60.73 ± 15.22	0.022
	< 40 cm/s (n=100)	77.51 ± 9.40		70.17 ± 13.53		69.52 ± 13.33	
**LA/Ao**	≥ 1.4 (n=66)	75.32 ± 10.19	0.015	69.00 ± 14.33	NS	65.66 ± 14.72	0.018
	< 1.4 (n=50)	79.84 ± 8.26		69.53 ± 13.40		72.07 ± 12.04	
	≥ 1.8 (n=18)	67.83 ± 10.20	< 0.001	61.39 ± 7.43	0.008	61.67 ± 14.89	0.028
	< 1.8 (n=98	79.00 ± 8.48		70.75 ± 12.66		69.58 ± 13.52	
	≥ 2 (n=4)	65.00 ± 6.06	0.010	50.75 ± 14.84	0.006	54.00 ± 15.43	0.037
	< 2 (n=112)	77.63 ± 9.49		69.92 ± 13.47		68.82 ± 13.73	
**LVEDD**[a]	≥ 3rd IQR (n=50)	76.35 ± 11.06	NS	66.86 ± 15.24	NS	67.80 ± 14.53	NS
	< 3rd IQR (n=53)	78.32 ± 8.11		71.72 ± 12.03		68.53 ± 12.51	

a. LVEDD obtained in 103 infants.

PDA, patent ductus arteriosus; LPA EDV, left pulmonary artery end-diastolic velocity; LA/Ao, left atrium-to-aortic root ratio; RSO2, regional oxyg en saturation; LVEDD, left ventricular end-diastolic diameter.
